# Pre-Treatment Physical Activity Could Positively Influence Pregnancy Rates in IVF despite the Induced Oxidative Stress: A Cohort Study on Salivary 8-Hydroxy-2′-deoxyguanosine

**DOI:** 10.3390/antiox11081586

**Published:** 2022-08-16

**Authors:** Viktória Prémusz, Dominika Lendvai-Emmert, Alexandra Makai, Krisztina Amrein, Shalini Chauhan, József Bódis, Kálmán András Kovács, Ákos Várnagy

**Affiliations:** 1 ELKH-PTE Human Reproduction Scientific Research Group, University of Pécs, H-7624 Pécs, Hungary; 2 Institute of Physiotherapy and Sport Sciences, Faculty of Health Sciences, University of Pécs, H-7621 Pécs, Hungary; 3 National Laboratory on Human Reproduction, University of Pécs, H-7622 Pécs, Hungary; 4 Doctoral School of Health Sciences, Faculty of Health Sciences, University of Pécs, H-7621 Pécs, Hungary; 5 Department of Neurosurgery, Medical School, University of Pécs, H-7624 Pécs, Hungary; 6 Neurotrauma Research Group, Szentágothai Research Centre, University of Pécs, H-7624 Pécs, Hungary; 7 Department of Obstetrics and Gynaecology, Medical School, University of Pécs, H-7624 Pécs, Hungary

**Keywords:** oxidative stress, fertilization in vitro, physical activity, DNA, 8-hydroxy-2′-deoxyguanosine

## Abstract

(1) Background: This study was designed to define whether pretreatment habitual physical activity (PA)-induced oxidative stress (OS) influences outcome measures by using 8-hydroxy-2′-deoxyguanosine (8-OHdG) in saliva samples of patients undergoing in vitro fertilization (IVF). (2) Method: In this cohort study, samples were obtained from 26 patients (age: 34.6 ± 5.5 years, BMI: 25.3 ± 5.1, infertility: 51.0 ± 28.7 months) before the treatment and a follow-up of outcome measures of IVF/ICSI. The 8-OHdG was evaluated by Abcam’s ELISA (ab201734), PA patterns by GPAQ-H and ActiGraph GT3X; (3) Results: The number of matured oocytes was positively influenced by the GPAQ-H recreation MET (R^2^ = 0.367, F = 10.994, *p* = 0.004; β = 0.005, *p* = 0.004, B Constant = 4.604) and a positive significant relationship (R^2^ = 0.757, F = 17.692, *p* < 0.001, B Constant = 1.342) was found with GPAQ-H recreational PA MET (β = 0.004, *p* < 0.001), and Grade 1 embryos and higher very vigorous activity (GT3X) were accompanied (R^2^ = 0.958, F = 408.479, *p* < 0.001) by higher ß-hCG levels (β = 63.703, *p* ≤ 0.001). Unanticipated positive correlation between 8-OHdG and ß-hCG level (R = 0.467, *p* = 0.028) was noticed, and there were significant differences in 8-OHdG in biochemical pregnancies (pregnant: 54.82 ± 35.56 ng/mL, non-pregnant: 30.06 ± 10.40 ng/mL, *p* = 0.022) as well. (4) Conclusions: Pretreatment PA could positively influence reproductive performance in IVF/ICSI despite the induced OS. However, a more sensitive biomarker and the recommended amount of activity should be further investigated.

## 1. Introduction

The benefits of regular physical activity in maintaining physical, mental, and social health are not called into question [1,2]. Depending on intensity or duration [3], certain studies controversially evaluate the health effects of exercise or even PA in general, including in relation to assisted reproductive treatments (ART) [4,5,6,7]. On the one hand, regular moderate-intensity exercise can induce the state of oxidative eustress by activating salient cell adaptive properties [8]. On the other hand, it can lead to an imbalance between the production and accumulation of oxygen reactive species (ROS) causing oxidative stress (OS) in cells and tissues [9,10,11] and to the detoxification capacity of biological systems for these reactive products [3]. An increase in ROS and free radicals production during exercise has both positive and negative physiological effects [12]. Even moderate exercise can increase ROS production, exceeding the capacity of antioxidant defenses [13,14]. 

As one of the main products of DNA oxidation, the intracellular concentration of 8-hydroxy-2′-deoxyguanosine (8-OHdG) could be a measure of OS. In the DNA strand, the hydroxyl radical (HO•) can interact with the nucleobases, such as guanine. This is how 8-OHdG is produced, which is a product of oxidative damage to 2′-deoxyguanosine in case ROS attacks the 8th carbon atom of guanine [15]. 

The role of OS in female reproduction has been broadly described [16,17,18,19]. An unequilibrium between ROS formation and the antioxidant defense system may mediate an excessive pathological process and negatively influence reproductive functions [20]. Extreme rates of ROS have adverse effects on female reproductive potential [21,22,23,24,25]. Preliminary reports have shown the correlation between ROS and metabolic adaptation in oocytes and embryos [26,27,28,29]. Their ability to tolerate OS has been shown to decrease, and metabolic activity has been shown to have a negative impact on their quality and development [30,31,32]. OS can affect cellular lipids, proteins, and DNA and cause cellular dysfunction, damage, or apoptosis. In serum, granulosa cells and FF 8-OHdG are negatively correlated with the quality of oocytes and embryos in in vitro fertilization (IVF) patients [15,33,34]. A study also revealed that higher 8-OHdG levels in follicular fluid was negatively correlated with intracytoplasmic sperm injection (ICSI) outcomes, and it is higher in nonpregnant women [29].

The present study was designed to define the effects of pretreatment habitual physical activity-induced oxidative stress on primary (pregnancy rates) and secondary (reproductive potential) outcomes by measuring 8-OHdG in saliva samples of patients undergoing IVF.

## 2. Materials and Methods

### 2.1. Patients

A single-center, observational cohort study was carried out at the assisted reproduction unit of a university-linked gynecological clinic in South Hungary. All female patients indicated for fertility treatment (IVF/ICSI) were invited to participate in the study with both female and male indications. Participants were consecutively recruited according to the date of the fertility consultation. The inclusion criteria were the following: BMI ≥ 18 kg/m^2^ and ≤38 kg/m^2^, and the absence of any significant health risk relevant to the IVF/ICSI procedure and outcome (metabolic and vascular diseases including diabetes mellitus, metabolic syndrome, fatty liver diseases and atherosclerosis, severe endometriosis (stage III or IV), and/or adenomyosis). Patients diagnosed with significant physical or mobility impairments would have been excluded, but no such patients entered the study.

**Figure 1:**
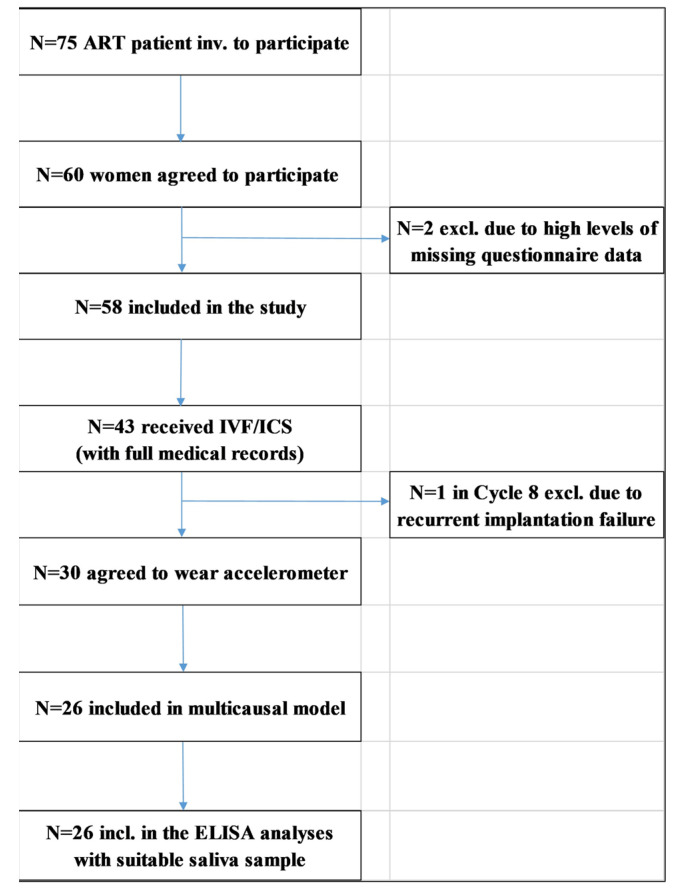
Flow diagram of patients’ enrolment.

Data collection was carried out during the routine examination and started on the 3rd day of the unstimulated cycles. A total of 75 women were invited and 60 of them agreed to participate in the study between December 2018 and June 2019, which means a 78.66% response rate. Primary outcomes of the IVF/ICSI were followed up between October 2019 and April 2020. In a conventional paper–pencil form, self-administered questionnaires were given to participants to fill out at home. Questionnaires were returned and saliva was provided on the 21st day of the unstimulated cycles. Due to high levels of missing data, 2 participants were excluded. Finally, 82.8% of the patients received IVF/ICSI and 30 agreed to wear accelerometers. Of these patients, 26 had suitable saliva samples and were included in the current analyses. The flow diagram of patients’ enrolment is presented in Figure 1.

The main characteristics of the study population are presented in Table 1 and Table 2. The mean age of the participants was 34.6 ± 5.5 years and their BMI ranged from 18.5 to 24.9 kg/m^2^. Higher education (50.0%), urban residence (69.2%), and active working (87.9%) were characteristic and almost all of them reported a satisfactory economic status (96.2%) (Table 1). These primarily nulliparous (84.6%) women were either married or lived with their partner (duration of the partnership: 7.6 ± 3.7 years) and had wanted to have a child for more than 4 years (51.0 ± 28.7 months), with mostly female infertility diagnoses (Table 2).

### 2.2. Laboratory Measurements

In human studies, 8-OHdG is measured primarily in the urine [35], but levels in the blood and saliva can also be determined. Due to the difficulty of storing urine samples, another easily collected, noninvasive human sample, saliva, was chosen to measure this biomarker [36]. The disadvantage of determining 8-OHdG in saliva may be that the level of this marker in saliva is lower, but the analytical methods obtained earlier values than other ELISA methods, but antibody cross-reactivity is common [37]. 

The 8-OHdG levels in saliva were measured using commercially available enzyme-linked immunosorbent assay (ELISA) kits from Abcam PLC (Cambridge, UK, ab201734), following the manufacturer’s instructions. The ELISA kit uses a highly sensitive monoclonal antibody against 8-OHdG with assay sensitivity of 0.59 ng/mL and with intra- and interassay coefficients of variation of ≤10%.

### 2.3. Collection of Saliva Samples

Saliva samples were taken from the patients before the stimulated cycle; the evening before (7 p.m.–9 p.m.) and in the morning—immediately after waking up (7 a.m.–9 a.m.)—of the 21st day of their menstrual cycles. About 1–1.5 mL of unstimulated saliva was collected by passive drool, using sterile Eppendorf tubes [38]. Sampling had to be performed before brushing teeth, at least 30 min after eating, drinking, chewing a gum, or playing sports, and after rinsing the mouth with water. Study participants were educated by a research assistant, then they collected their samples at home and recorded the exact time (hh: mm) of sampling. After sampling, saliva samples were stored in a refrigerator at 2–8 degrees Celsius for up to 72 h, after which the samples were prepared by centrifugation at 3000 rpm for 5 min. After supernatant aspiration, samples were stored at −20 °C for approximately one month until the 8-OHdG level was determined [39].

### 2.4. Protocols

The inclusion of patients in IVF/ICSI procedures was decided/approved by two independent physicians. Indices of reproductive potential were described with the number of oocytes (retrieved and matured), embryos (Grade 1), and the level of serum beta-human chorionic gonadotropin (β-hCG) on day 12 (biochemical pregnancy) as secondary outcomes or with clinical pregnancy as the primary outcome of IVF/ICSI. The protocol of controlled ovarian hyperstimulation and fertilization was presented in our previous publication [34]. Grade 1 staged embryos were transferred according to the consensus embryo scoring system of ESHRE [40]. To evaluate the success of the treatment, the ß-hCG level was tested on day 12 (biochemical pregnancy) and the gestational sac was detected by transvaginal ultrasound examination 21 days after the embryo transfer (clinical pregnancy) [41].

Both in the short and long protocols, triptorelin (Gonapeptyl, Ferring, Germany) by GnRH agonist, and cetrorelix (Cetrotide, Merck, Europe) by antagonist protocol were used to induce IVF/ICSI. Depending on the follicular maturation, the stimulation was performed with an individual dosage of rFSH (Gonal-F; Serono Aubonne, Switzerland) varying from 100 to 225 IU per day, adapted to the BMI and the age and with an increased maximum dose of 300–350 IU per day for low-response patients. No significant difference was detected in the cumulative dosage of GnRH or rFSH between women with or without chemical/clinical pregnancy.

### 2.5. Assessment Scales

Participants were asked to answer questions regarding age, educational level, income, marital status, the duration of the partnership, the duration of infertility, BMI, and lifestyle habits to describe main characteristics.

To define PA and exercise habits, participants self-reported exercise intensity and frequency and all types of PA. WHO’s PA questionnaire, the Global Physical Activity Questionnaire (GPAQ), was applied [42,43,44,45]. This multidomain questionnaire contains 16 items on intensity, frequency, and duration in three fields of PA: work, active transportation, recreation or leisure time, and sedentary behavior in the past week. Data represent time (minutes) spent with PA or energy expenditure (MET: Metabolic Equivalent of Task). The Hungarian version of GPAQ proved to be reliable and valid in our previous study [46]. In addition to GPAQ, participants were asked to self-report their exercise in a PA diary, detailing its type and frequency.

### 2.6. Assessment of Physical Activity

Self-reports were compared with objective measures. Triaxial ActiGraph GT3X+ accelerometers (ActiGraph, Pensacola, FL, USA) were used with standard device initialization (30 Hz sample rate, 60-s epochs, normal filter option). It measures the strength and duration of movements in three spatial directions and converts the acceleration into a digital signal that can be quantified and measured. Participants were trained by a research assistant on wearing the accelerometer. The devices were worn on the right hip for one week during all waking hours, excluding any water-based activities or contact sports; ≥60 min without movement was defined as “nonwear time”. At least 8 h of daily wear-time and 5–7 valid days were required for inclusion [47]. ActiLife 6 software and Freedson cut-points were used for the analyses [48]. The average of daily moderate to vigorous physical activity (MVPA) (min/day) and sedentary behavior (SB) (min/day) was calculated [49] with a sensitivity and specificity of more than 98% and 99%, respectively [50]. Participants gave the accelerometers back on the 8th day, which was the 21st day of their menstrual cycle, and the day of saliva sampling. 

### 2.7. Data Analysis

Statistical analyses were performed using SPSS 26.0 software (SPSS Inc., Chicago, IL, USA). Kolmogorov–Smirnov test was conducted for normality testing. To compare continuous variables, Mann–Whitney U-test was applied. To test the association between two continuous variables, Spearman’s rank correlation was used. To define predicting factors of primary and secondary outcomes of IVF from pretreatment habitual PA, psycho-socio-demographic, and baseline biomedical variables, multivariate linear regression with a stepwise method was conducted. Data were expressed as mean ± standard deviation or as a number (percentage) and the level of significance level was set at *p* ˂ 0.05.

## 3. Results

A description of PA patterns, such as intensity, frequency, and mode are shown in Table 3. A total of 57.14% of the respondents reported substantial leisure-time PA, and only 40.48% met the requirement of 150 min/week of moderate physical activity. They self-reported 20.8 h of activity per week and 6 h of sitting per day (Table 3).

Based on the primary outcomes, the women were divided into two groups. Exerciselike activities were more typical of women who became pregnant, spending more time and energy on recreational or vigorous PA. Women who failed to conceive had higher PA values compared to work or overall. However, these differences were not significant. Examining the secondary outcomes, we could describe a significant relationship with some markers of reproductive potential, expressed by the number of retrieved and matured oocytes (R = 0.315, *p* = 0.045; R = 0.339, *p* = 0.030, respectively). Regarding Grade 1 embryos, the relationship was not significant. The results were applicable for patients who reached a minimum of 150 min of RPA based on their self-reports (GPAQ-H) (Table 4).

To define predicting factors of secondary outcomes of IVF (such as the number of matured oocytes and embryos, as well as the level of ß-hCG on day 12), concerning PA and OS, a multivariate linear regression using the stepwise method was conducted. Results were adjusted for education, BMI, age, duration of child-wish, duration of infertility, number of cycles, objective and subjective PA, and OS as confounders. In Model 1, the number of oocytes was positively influenced by the GPAQ-H recreation MET (R^2^ = 0.367, F = 10.994, *p* = 0.004; β = 0.005, *p* = 0.004, B Constant = 4.604). In Model 2 (R^2^ = 0.757, F = 17.692, *p* < 0.001, B Constant = 1.342), a positive significant relationship was found with GPAQ-H recreational physical activity MET (β = 0.004, *p* < 0.001) and a negative relationship with BMI (β = −0.167, *p* = 0.038). In Model 3 (R^2^ = 0.958, F = 408.479, *p* < 0.001), higher very vigorous activity (GT3X) was accompanied by higher ß-hCG levels (β = 63.703, *p* ≤ 0.001). 

**Figure 2:**
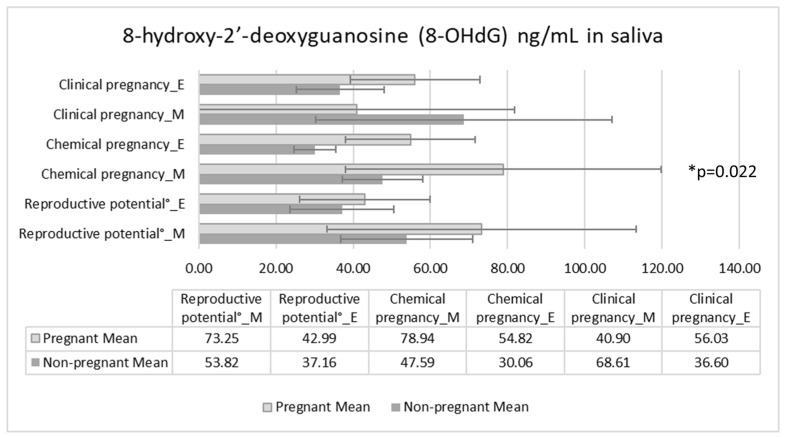
Differences between the 8-hydroxy-2′-deoxyguanosine (8-OHdG) ng/mL levels in patients undergoing IVF using saliva samples. E: evening, M: morning, number of matured oocytes over 6. * *p* ˂ 0.05.

Regarding OS, there was no detectable relationship with reproductive potential, which is determined by the number of retrieved oocytes, mature oocytes, and Grade 1 embryos. Nevertheless, we found a significant positive correlation with Spearman’s rank correlation analysis between 8-OHdG and ß-hCG levels (R = 0.467, *p* = 0.028). Results showed significant elevation in 8-OHdG morning samples in biochemical pregnancies, measured with the Mann–Whitney U-test (pregnant: 54.82 ± 35.56 ng/mL, nonpregnant: 30.06 ± 10.40 ng/mL, *p* = 0.022). An unexpected circadian difference was found between morning and evening samples, the level of 8-OHdG in morning samples was significantly higher except for clinical pregnancy. In the pregnant group, we could not observe the peak after waking up; however, there was a decrease in the morning samples (Figure 2).

Despite the above relationships, no correlation could be found between the types and intensity of PA and the level of OS (Table 5).

## 4. Discussion

The incidence of assisted reproductive therapies is increasing, but their success rates have not risen significantly in the last decade, which highlights the need for a detailed examination of lifestyle covariates, especially physical activity [51,52]. Our previous cohort study, [53] with a follow-up of primary and secondary outcomes [54], suggests that an abundance of pretreatment PA may support the course of IVF/ICSI and increase its success rate. Similar to the current study, a significant positive relationship with reproductive potential could only be described in women who achieved at least 150 min of recreational type of PA. However, the upper limit of the appropriate PA could not be determined. It was assumed that examining PA-induced OS processes, such as possible negative consequences of strenuous exercise, could help define the optimal duration and intensity of PA.

In our study population, women who succeeded to conceive and get pregnant (primary outcome) spent more time and energy expenditure on exercise-like activities (recreational or vigorous PA), while work-related PA was more typical in cases of failure. A positive relationship was found between RPA of at least 150 min per week prior to the treatment and reproductive potential (secondary outcomes) based on the number of retrieved oocytes, mature oocytes, and Grade 1 embryos, and higher very vigorous activity (which could also reflect on exercise-like activities) was accompanied by higher ß-hCG levels. Contrary to the above findings, an unanticipated positive correlation was observed between 8-OHdG and ß-hCG levels and there were significant differences in 8-OHdG in biochemical pregnancies as well. An unexpected circadian difference was found between morning and evening samples, the level of 8-OHdG was significantly higher in morning samples, except for clinical pregnancy. In the pregnant group, instead of the morning peak, the level of 8-OHdG decreased.

The review of Kawamura et al. focused on the evidence of OS caused by acute exercise in organisms, mostly in healthy individuals, and agreed with the consensus that exercise increases the production of free radicals. Direct measurement is difficult due to high reactivity and extremely short half-lives, which is why measurement of OS markers, including oxidation products, is preferred as indicators. However, they proposed to assess them by measuring multiple markers [55]. 

Powers and Jackson classified biomarkers related to in vivo OS into the following categories: free radicals (ROS), antioxidant levels in tissues, oxidation products, and redox balance. They considered the third one, the oxidation products, to be the most important aspect in OS assessment, including protein carbonyl (PC), a marker of protein oxidation; F2-isoprostanes and malondialdehyde (MDA), markers of lipid peroxidation; and 8-OHdG, a marker of deoxyribonucleic acid (DNA) oxidation [56]. The biomarker 8-OHdG has been considered a sensitive indicator of oxidative DNA damage [57,58,59,60]. 

The study of Llorente-Cantarero described the effect of PA and SB objectively measured by ActiGraphs on the oxidative stress state of children and adolescents. They used the PASS index integrating main domains of the WHO recommendations to evaluate the health benefits of increased physical activity and reduced sedentary time. In this study, lower urinary 8-OHdG was associated with higher PASS (UNIANCOVA *p* = 0.005) showing a better redox profile. They noted that to achieve redox homeostasis, reduced OS with higher PASS may diminish the need to maintain high concentrations of antioxidants in plasma during rest [61].

The biomarker 8-OHdG was also used for the assessment of exercise-induced oxidative damage in adult populations in several studies. No firm relationship between exercise and oxidative damage can be described, but there is a tendency of increased 8-OHdG levels during extensive exercise. In contrast to children, physically active middle-aged subjects had consistently higher urinary 8-OHdG levels than sedentary individuals [62,63,64].

The beneficial health effects of OS seem, at first, inconsistent. To address the above incoherent results, Sasaki et al. continued the research and found higher oxidative as well as IgM response to reactive carbonyl derivatives, which could provide a basis for health benefits in active middle-aged subjects [64]. Kazuaki Kawai and coauthors postulated that salivary 8-hydroxyguanine (8-OHGua) may be a useful noninvasive and promising oxidative stress biomarker instead of urinary because it is easier to collect. They found significantly higher (*p* = 0.013) levels in healthy smoker subjects than in nonsmokers (3.80 ng/mL) with HPLC-ECD [35].

Seino et al. addressed the issue of OS responses in women during IVF and described 8-OHdG expression in granulosa cells and its negative effects on the quality of oocytes/embryos and primary outcome [33]. Karuputhula et al. found that increased ROS generation was associated with increased DNA damage in the granulosa cells of IVF patients with endometriosis [65]. Tamura et al. measured 8-OHdG in follicular fluid (FF) and published a significant correlation between elevated concentrations and higher rates of oocyte degeneration. Controversially, melatonin was also found to protect oocytes from oxidative damage and to improve reproductive performance [15].

In a previous study by our team, the two OS markers TAC and 8-OHdG were measured in maternal serum and FF of women undergoing IVF. Consistent with the results of Seino [33] and Tamura [15], serum levels were inversely related and higher TAC and 8-OHdG levels in FF negatively affected the number of high-quality embryos. 

The review of Kawamura et al., based on different studies, pointed out a Janus-faced process: the regular exercise may affect exercise-induced OS by increasing antioxidant levels, but long-term exercise training may reduce OS following acute exercise [66]. However, it should be noted that these studies usually measure athletes or work with highly exhausting protocols. For example, one of these training programs consisted of running at 80% maximal exercise heart rate for 60 min daily, 5 days per week for 12 weeks [12,66]. The study of Gudmundsdottir et al. measured the effects of PA on female fertility in the general population and demonstrated that women who were active most days experienced fertility problems 3.2 times more often. Based on the study, exercise to the point of exhaustion also appeared to be harmful, leading to 2.3 times more fertility impairments than low-intensity PA [67]. Level II-2 evidence proves the significance of lifetime exercise in the study of Morris et al. Women who exercised 4 h or more per week were 40% less likely to have a successful live birth compared to those who did not exercise (OR 0.6, CI 0.4–0.8). This was three times more likely to lead to cycle interruption (OR 2.8, CI 1.5–5.3) and twice as likely to lead to implantation failure or pregnancy loss (OR 2.0, CI 1.4–3.1; OR 2.0, CI 1.2–3.4, respectively) [7]. 

We paid special attention to those participants who reported at least 4 h of exercise per week (18.2%) in our study. However, neither negative nor positive effects were detected in case of activity exceeding 240 or even 300 min per week. However, regarding reproductive potential (number of retrieved and matured oocytes), a significant relationship could be described in those participants who achieved an RPA of at least 150 min prior to the treatment as measured by GPAQ-H.

## 5. Conclusions

It is widely accepted that all exercise causes oxidative stress, which leads to the induction of an antioxidative response and, in the long term, leads to an improvement in the ability to develop an antioxidative response. 

OS contributed significantly to reproductive potential as well as pregnancy rates in IVF/ICSI. Our present study suggests that pretreatment PA may also positively influence reproductive potential during IVF/ICSI. 

The importance of special forms of exercise, in addition to daily physical activity, proposes the development of a specific, pretreatment intervention program for IVF/ICSI participants. However, the ideal biomarkers need to be further explored to define the optimum level of PA. 

The limitations of the study include the modest sample size and its nonrepresentative nature. To obtain more convincing results beyond detailed objective assessment of physical activity, an increased number of participants in a well-powered randomized, controlled prospective study is needed. To verify the results, more data on DNA quality, antral follicle count, and antimullerian hormone levels would have been beneficial. To quantify physical activity-induced oxidative stress, an integrative, multimarker panel would show more reliable results.

## Figures and Tables

**Table 1:** Socio-demographic, health status, and lifestyle characteristics of the patients studied (N = 26).

Socio-Demographic Data ^1^	
**Age (years)**	34.6 ± 5.5
**Education**	
Low	5 (19.2%)
Intermediate	8 (30.8%)
High	13 (50.0%)
**Marital status**	
Married	17 (65.4%)
Partner	9 (34.6%)
**Place of residence**	
County seat	8 (30.8%)
City	10 (38.5%)
Village	8 (30.8%)
**Income**	
Low	1 (3.8%)
Medium	16 (61.5%)
High	9 (34.6%)
**Health Status and Lifestyle**	
**BMI (kg/m^2^)**	25.3 ± 5.1
Underweight (<18.5)	0 (0.0%)
Normal weight (18.5–24.9)	18 (69.2%)
Overweight (25–29.9)	2 (7.7%)
Obesity (>30)	6 (23.1%)
**Self-Rated Physical Health**	
Poor	0 (0.0%)
Fair	0 (0.0%)
Neither good nor bad	5 (19.2%)
Good	16 (61.5%)
Excellent	5 (19.2%)
**Healthy Diet**	
Pay attention	25 (96.2%)
Not really/No attention	1 (3.8%)
**Tobacco Use**	
Nonsmoker	26 (100%)
**Exercise**	
Regularly	12 (46.2%)
Not	14 (53.8%)

^1^ Data expressed as mean ± SD or N (%), BMI: body mass index.

**Table 2:** Diagnosis and treatment history of the patients studied (N = 26).

Diagnosis and Treatment History ^1^	
**Medical indication**	
Poor semen quality	6 (23.1%)
Fallopian tube pathology	8 (30.8%)
Endometriosis	4 (15.4%)
Other female indication	1 (3.8%)
Unexplained	7 (26.9%)
**Child-wish (months)**	51.0 ± 28.7
**Relationship (years)**	6.7 ± 3.6
**Gravidity**	
Nulligravid	14 (53.8%)
**Parity**	
Nulliparous	22 (84.6%)
**IVF/ICSI Procedures**	2.0 ± 0.1
Cycle 1	9 (34.6%)
Cycle 2	11 (44.0%)
Cycle 3	3 (12.0%)
Cycle 4	3 (12.0%)
**Laboratory measures**	
Serum estradiol—pmol/L	2007.7 ± 2702.0
FSH *—IU	2196.2 ± 524.4
Gonadotropin *—IU	0.9 ± 0.2
**Secondary outcome**	
No. of oocytes retrieved	7.5 ± 5.4
No. of matured oocytes **	5.1 ± 4.3
No. of Grade 1 embryos	3.4 ± 3.4
No. of transferred embryos	1.4 ± 0.9
ß-hCG on day 12—IU	165.9 ± 352.5
**Primary outcome**	
Clinical pregnancy	4 (15.38%)
Biochemical pregnancy	9 (34.6%)
**8OHdG in saliva**	
Morning sample (ng/mL)	61.9 ± 64.6
Evening sample (ng/mL)	39.6 ± 27.2

^1^ Data expressed as mean ± SD or N (%). FSH: follicle-stimulating hormone. ICSI: intracytoplasmic sperm injection. IVF: in vitro fertilization. hCG: human chorionic gonadotropin. SD: standard deviation. 8OHdG: 8-hydroxy-2′-deoxyguanosine. * Total dose administrated. ** Metaphase II.

**Table 3:** Pretreatment physical activity characteristics of women undergoing IVF/ICSI by GPAQ-H questionnaires and ActiGraph GT3X data (N = 26).

GPAQ-H		
Measure	Mean	SD
Work—VPA		
min/week	287.31	610.83
MET	1723.85	3665.00
Work—MPA		
min/week	566.54	623.50
MET	1699.62	1870.50
Transport		
min/week	292.88	483.35
MET	878.65	1450.04
Recreation—VPA		
min/week	24.23	54.35
MET	145.38	326.07
Recreation—MPA		
min/week	76.54	126.95
MET	229.62	380.86
Sitting		
min/week	2487.69	1655.35
Total MVPA		
min/week	954.62	968.79
MET	4677.12	4835.86
**ActiGraph GT3X**		
Measure (min/week)	Mean	SD
Sedentary	8169.05	1328.43
Light	1191.23	381.43
Moderate	209.81	145.29
Vigorous	1.34	1.66
Very Vigorous	0.33	1.20
Total MVPA	211.16	146.95

GPAQ-H: Global Physical Activity Questionnaire—Hungarian version, MET: metabolic equivalent of task, MPA: moderate physical activity, MVPA: moderate to vigorous physical activity, VPA: vigorous physical activity.

**Table 4:** Correlation between recreational physical activity (RPA) measured by GPAQ-H and secondary outcomes of IVF/ICSI by women who reached at least 150 min RPA.

Secondary Outcomes	R	*p*
Retrieved oocytes	0.315	0.045 *
Matured oocytes	0.339	0.030 *
Grade 1 embryos	0.294	0.062

GPAQ-H: Global Physical Activity Questionnaire—Hungarian version, R: correlation coefficient for Spearman’s rho, *p*: level of significance, RPA: recreational physical activity, ICSI: intracytoplasmic sperm injection, IVF: in vitro fertilization, * *p* ˂ 0.05.

**Table 5:** Correlation between the types (work, active transport, recreation) and intensity of physical activity based on subjective and objective measures (GPAQ-H and ActiGraph GT3X, min/week) and the level of oxidative stress measured with saliva 8-OHdG (N = 26).

Correlation with Saliva 8-OHdG		
**GPAQ-H**	Morning sample (R)	Evening sample (R)
Work—VPA	0.243	−0.123
Work—MPA	0.167	0.209
Transport	−0.010	−0.009
Recreation—VPA	0.162	−0.031
Recreation—MPA	0.269	0.060
Total—MVPA	0.207	0.064
**ActiGraph**		
Sedentary	0.346	0.271
Light	0.232	−0.300
Moderate	0.211	−0.150
Vigorous	−0.027	−0.283
Very Vigorous	−0.404	−0.036
Total MVPA	0.168	−0.100

GPAQ: Global Physical Activity Questionnaire, MPA: moderate physical activity, MVPA: moderate to vigorous physical activity, R: correlation coefficient for Spearman’s rho, the level of significance was *p* > 0.05 for every correlation, VPA: vigorous physical activity, 8OHdG: 8-hydroxy-2′-deoxyguanosine.

## Data Availability

Data are available from the authors upon reasonable request.
